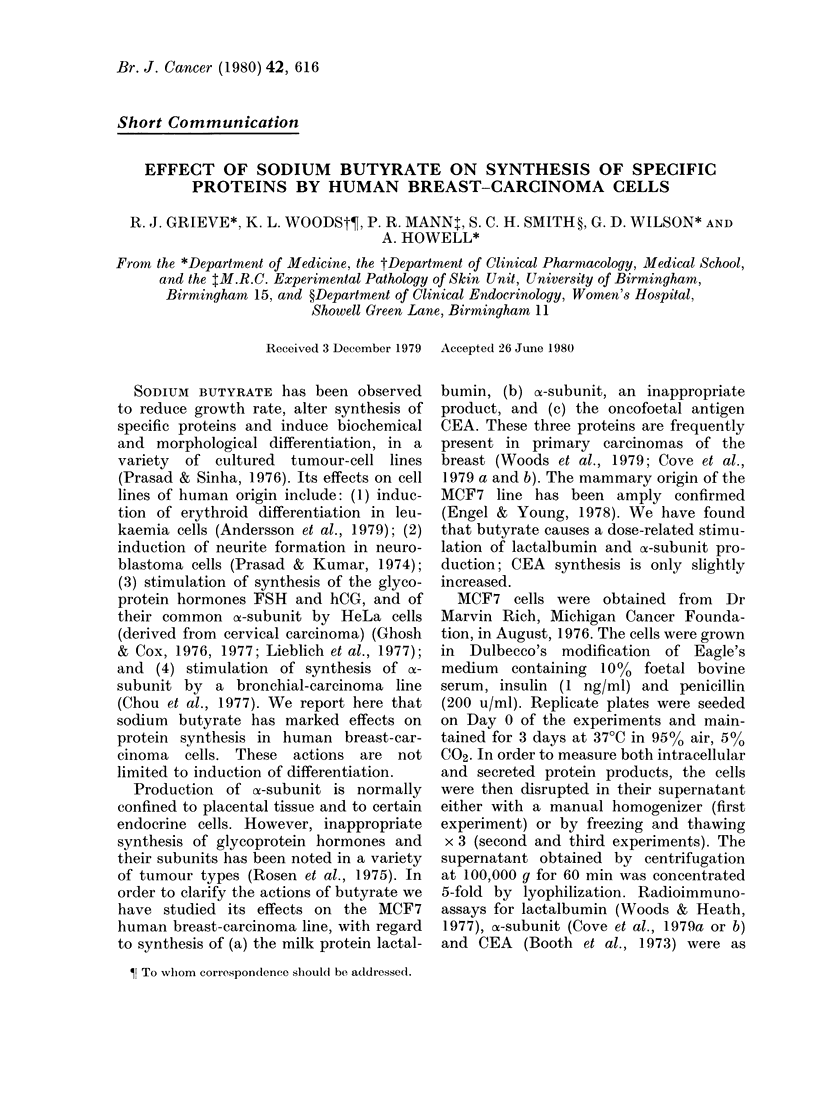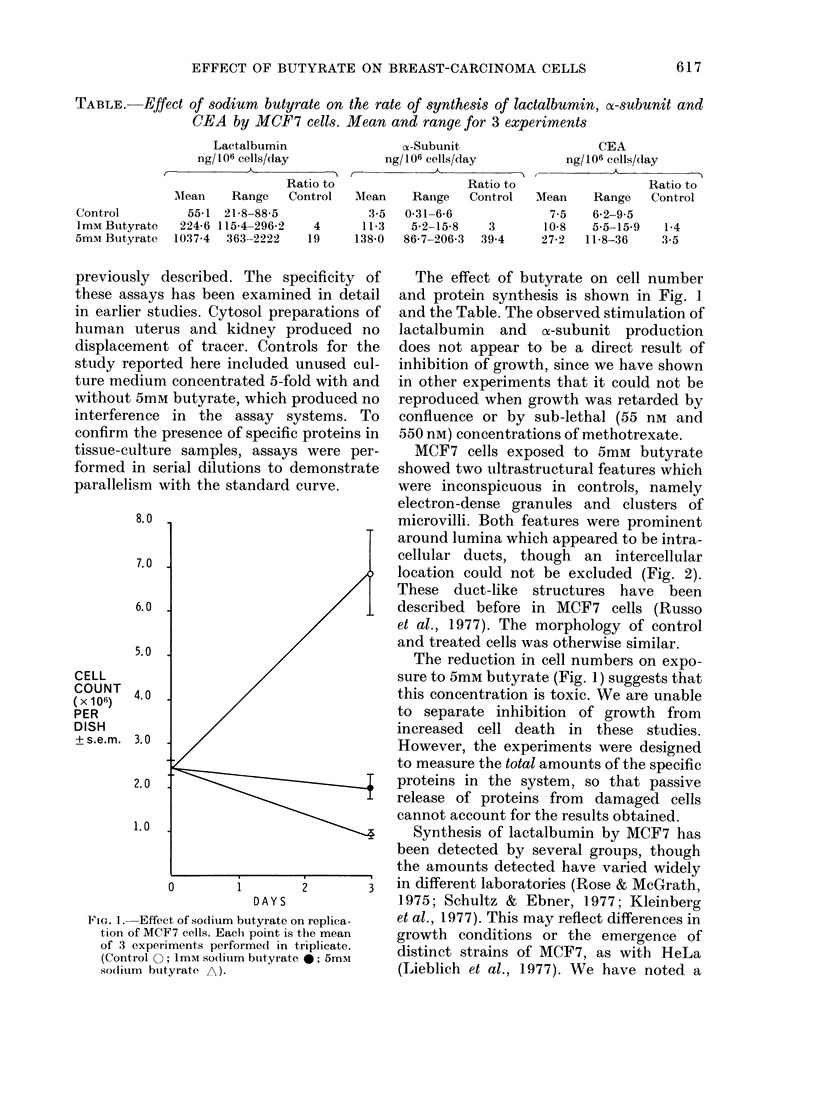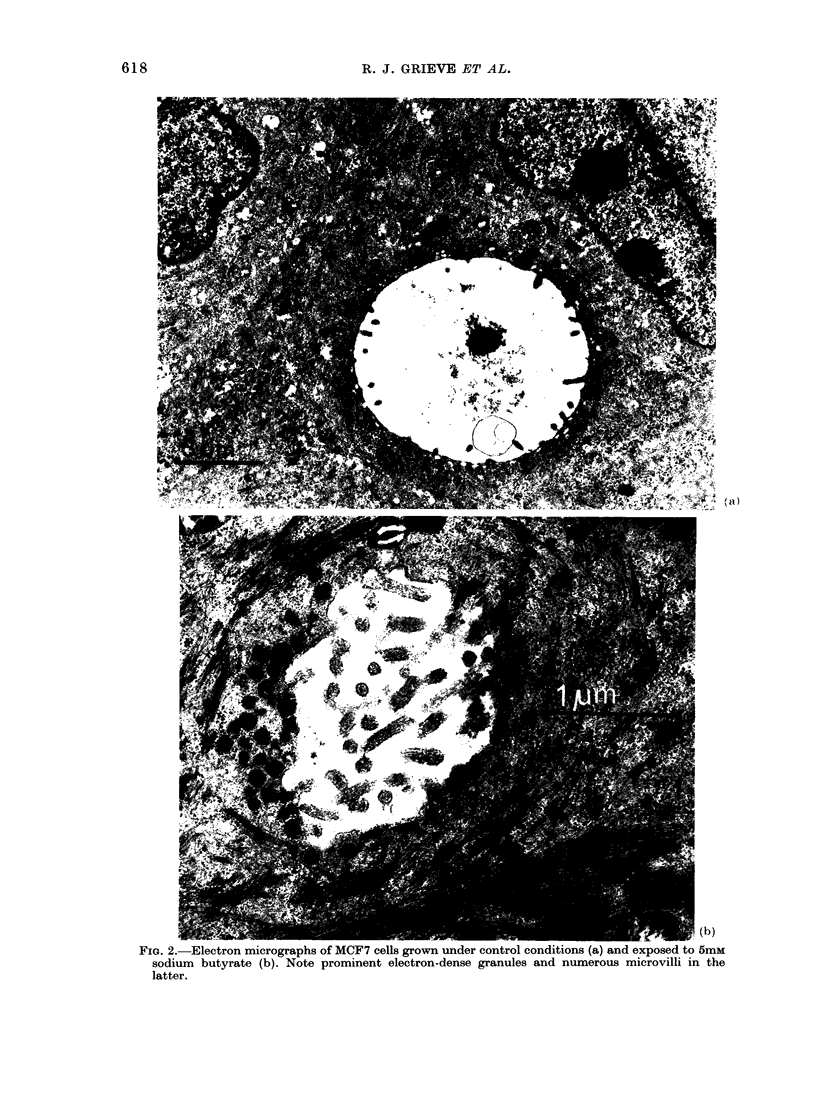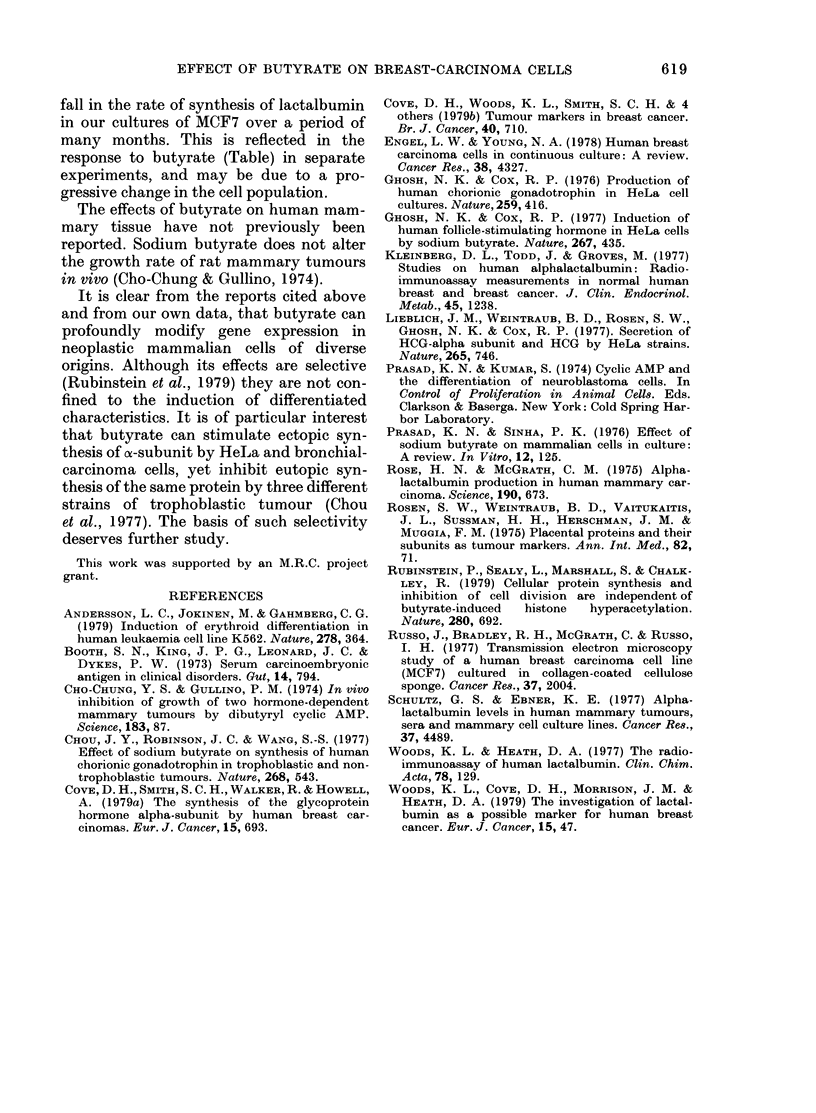# Effect of sodium butyrate on synthesis of specific proteins by human breast-carcinoma cells.

**DOI:** 10.1038/bjc.1980.287

**Published:** 1980-10

**Authors:** R. J. Grieve, K. L. Woods, P. R. Mann, S. C. Smith, G. D. Wilson, A. Howell

## Abstract

**Images:**


*Br. J. Cancer* (1980) **42**, 616

*Short Communication*

### EFFECT OF SODIUM BUTYRATE ON SYNTHESIS OF SPECIFIC PROTEINS BY HUMAN BREAST-CARCINOMA CELLS

R. J. GRIEVE*, K. L. WOODS†¶, P. R. MANN‡, S. C. H. SMITH§, G. D. WILSON* AND A. HOWELL*

*From the *Department of Medicine, the †Department of Clinical Pharmacology, Medical School, and the ‡M.R.C. Experimental Pathology of Skin Unit, University of Birmingham, Birmingham* 15, *and §Department of Clinical Endocrinology, Women's Hospital, Showell Green Lane, Birmingham* 11

Received 3 December 1979 Accepted 26 June 1980

SODIUM BUTYRATE has been observed to reduce growth rate, alter synthesis of specific proteins and induce biochemical and morphological differentiation, in a variety of cultured tumour-cell lines (Prasad & Sinha, 1976). Its effects on cell lines of human origin include: (1) induction of erythroid differentiation in leukaemia cells (Andersson *et al.*, 1979); (2) induction of neurite formation in neuroblastoma cells (Prasad & Kumar, 1974); (3) stimulation of synthesis of the glycoprotein hormones FSH and hCG, and of their common α-subunit by HeLa cells (derived from cervical carcinoma) (Ghosh & Cox, 1976, 1977; Lieblich *et al.*, 1977); and (4) stimulation of synthesis of α-subunit by a bronchial-carcinoma line (Chou *et al.*, 1977). We report here that sodium butyrate has marked effects on protein synthesis in human breast-carcinoma cells. These actions are not limited to induction of differentiation.

Production of α-subunit is normally confined to placental tissue and to certain endocrine cells. However, inappropriate synthesis of glycoprotein hormones and their subunits has been noted in a variety of tumour types (Rosen *et al.*, 1975). In order to clarify the actions of butyrate we have studied its effects on the MCF7 human breast-carcinoma line, with regard to synthesis of (a) the milk protein lactalbumin, (b) α-subunit, an inappropriate product, and (c) the oncofoetal antigen CEA. These three proteins are frequently present in primary carcinomas of the breast (Woods *et al.*, 1979; Cove *et al.*, 1979 *a* and *b*). The mammary origin of the MCF7 line has been amply confirmed (Engel & Young, 1978). We have found that butyrate causes a dose-related stimulation of lactalbumin and α-subunit production; CEA synthesis is only slightly increased.

MCF7 cells were obtained from Dr Marvin Rich, Michigan Cancer Foundation, in August, 1976. The cells were grown in Dulbecco's modification of Eagle's medium containing 10% foetal bovine serum, insulin (1 ng/ml) and penicillin (200 u/ml). Replicate plates were seeded on Day 0 of the experiments and maintained for 3 days at 37°C in 95% air, 5% $CO_2$. In order to measure both intracellular and secreted protein products, the cells were then disrupted in their supernatant either with a manual homogenizer (first experiment) or by freezing and thawing ×3 (second and third experiments). The supernatant obtained by centrifugation at 100,000 *g* for 60 min was concentrated 5-fold by lyophilization. Radioimmunoassays for lactalbumin (Woods & Heath, 1977), α-subunit (Cove *et al.*, 1979*a* or *b*) and CEA (Booth *et al.*, 1973) were as

¶ To whom correspondence should be addressed.

TABLE.—*Effect of sodium butyrate on the rate of synthesis of lactalbumin, α-subunit and CEA by MCF7 cells. Mean and range for 3 experiments*

| | Lactalbumin ng/$10^6$ cells/day | | | α-Subunit ng/$10^6$ cells/day | | | CEA ng/$10^6$ cells/day | | |
|---|---|---|---|---|---|---|---|---|---|
| | Mean | Range | Ratio to Control | Mean | Range | Ratio to Control | Mean | Range | Ratio to Control |
| Control | 55·1 | 21·8–88·5 | | 3·5 | 0·31–6·6 | | 7·5 | 6·2–9·5 | |
| 1mM Butyrate | 224·6 | 115·4–296·2 | 4 | 11·3 | 5·2–15·8 | 3 | 10·8 | 5·5–15·9 | 1·4 |
| 5mM Butyrate | 1037·4 | 363–2222 | 19 | 138·0 | 86·7–206·3 | 39·4 | 27·2 | 11·8–36 | 3·5 |

previously described. The specificity of these assays has been examined in detail in earlier studies. Cytosol preparations of human uterus and kidney produced no displacement of tracer. Controls for the study reported here included unused culture medium concentrated 5-fold with and without 5mM butyrate, which produced no interference in the assay systems. To confirm the presence of specific proteins in tissue-culture samples, assays were performed in serial dilutions to demonstrate parallelism with the standard curve.

FIG. 1.—Effect of sodium butyrate on replication of MCF7 cells. Each point is the mean of 3 experiments performed in triplicate. (Control ○; 1mM sodium butyrate ●; 5mM sodium butyrate △).

The effect of butyrate on cell number and protein synthesis is shown in Fig. 1 and the Table. The observed stimulation of lactalbumin and α-subunit production does not appear to be a direct result of inhibition of growth, since we have shown in other experiments that it could not be reproduced when growth was retarded by confluence or by sub-lethal (55 nM and 550 nM) concentrations of methotrexate.

MCF7 cells exposed to 5mM butyrate showed two ultrastructural features which were inconspicuous in controls, namely electron-dense granules and clusters of microvilli. Both features were prominent around lumina which appeared to be intracellular ducts, though an intercellular location could not be excluded (Fig. 2). These duct-like structures have been described before in MCF7 cells (Russo *et al.*, 1977). The morphology of control and treated cells was otherwise similar.

The reduction in cell numbers on exposure to 5mM butyrate (Fig. 1) suggests that this concentration is toxic. We are unable to separate inhibition of growth from increased cell death in these studies. However, the experiments were designed to measure the *total* amounts of the specific proteins in the system, so that passive release of proteins from damaged cells cannot account for the results obtained.

Synthesis of lactalbumin by MCF7 has been detected by several groups, though the amounts detected have varied widely in different laboratories (Rose & McGrath, 1975; Schultz & Ebner, 1977; Kleinberg *et al.*, 1977). This may reflect differences in growth conditions or the emergence of distinct strains of MCF7, as with HeLa (Lieblich *et al.*, 1977). We have noted a

(a)

(b)

FIG. 2.—Electron micrographs of MCF7 cells grown under control conditions (a) and exposed to 5mM sodium butyrate (b). Note prominent electron-dense granules and numerous microvilli in the latter.

fall in the rate of synthesis of lactalbumin in our cultures of MCF7 over a period of many months. This is reflected in the response to butyrate (Table) in separate experiments, and may be due to a progressive change in the cell population.

The effects of butyrate on human mammary tissue have not previously been reported. Sodium butyrate does not alter the growth rate of rat mammary tumours *in vivo* (Cho-Chung & Gullino, 1974).

It is clear from the reports cited above and from our own data, that butyrate can profoundly modify gene expression in neoplastic mammalian cells of diverse origins. Although its effects are selective (Rubinstein *et al.*, 1979) they are not confined to the induction of differentiated characteristics. It is of particular interest that butyrate can stimulate ectopic synthesis of α-subunit by HeLa and bronchial-carcinoma cells, yet inhibit eutopic synthesis of the same protein by three different strains of trophoblastic tumour (Chou *et al.*, 1977). The basis of such selectivity deserves further study.

This work was supported by an M.R.C. project grant.

REFERENCES

ANDERSSON, L. C., JOKINEN, M. & GAHMBERG, C. G. (1979) Induction of erythroid differentiation in human leukaemia cell line K562. *Nature*, **278**, 364.

BOOTH, S. N., KING, J. P. G., LEONARD, J. C. & DYKES, P. W. (1973) Serum carcinoembryonic antigen in clinical disorders. *Gut*, **14**, 794.

CHO-CHUNG, Y. S. & GULLINO, P. M. (1974) *In vivo* inhibition of growth of two hormone-dependent mammary tumours by dibutyryl cyclic AMP. *Science*, **183**, 87.

CHOU, J. Y., ROBINSON, J. C. & WANG, S.-S. (1977) Effect of sodium butyrate on synthesis of human chorionic gonadotrophin in trophoblastic and non-trophoblastic tumours. *Nature*, **268**, 543.

COVE, D. H., SMITH, S. C. H., WALKER, R. & HOWELL, A. (1979*a*) The synthesis of the glycoprotein hormone alpha-subunit by human breast carcinomas. *Eur. J. Cancer*, **15**, 693.

COVE, D. H., WOODS, K. L., SMITH, S. C. H. & 4 others (1979*b*) Tumour markers in breast cancer. *Br. J. Cancer*, **40**, 710.

ENGEL, L. W. & YOUNG, N. A. (1978) Human breast carcinoma cells in continuous culture: A review. *Cancer Res.*, **38**, 4327.

GHOSH, N. K. & COX, R. P. (1976) Production of human chorionic gonadotrophin in HeLa cell cultures. *Nature*, **259**, 416.

GHOSH, N. K. & COX, R. P. (1977) Induction of human follicle-stimulating hormone in HeLa cells by sodium butyrate. *Nature*, **267**, 435.

KLEINBERG, D. L., TODD, J. & GROVES, M. (1977) Studies on human alphalactalbumin: Radioimmunoassay measurements in normal human breast and breast cancer. *J. Clin. Endocrinol. Metab.*, **45**, 1238.

LIEBLICH, J. M., WEINTRAUB, B. D., ROSEN, S. W., GHOSH, N. K. & COX, R. P. (1977). Secretion of HCG-alpha subunit and HCG by HeLa strains. *Nature*, **265**, 746.

PRASAD, K. N. & KUMAR, S. (1974) Cyclic AMP and the differentiation of neuroblastoma cells. In *Control of Proliferation in Animal Cells*. Eds. Clarkson & Baserga. New York: Cold Spring Harbor Laboratory.

PRASAD, K. N. & SINHA, P. K. (1976) Effect of sodium butyrate on mammalian cells in culture: A review. *In Vitro*, **12**, 125.

ROSE, H. N. & MCGRATH, C. M. (1975) Alpha-lactalbumin production in human mammary carcinoma. *Science*, **190**, 673.

ROSEN, S. W., WEINTRAUB, B. D., VAITUKAITIS, J. L., SUSSMAN, H. H., HERSCHMAN, J. M. & MUGGIA, F. M. (1975) Placental proteins and their subunits as tumour markers. *Ann. Int. Med.*, **82**, 71.

RUBINSTEIN, P., SEALY, L., MARSHALL, S. & CHALKLEY, R. (1979) Cellular protein synthesis and inhibition of cell division are independent of butyrate-induced histone hyperacetylation. *Nature*, **280**, 692.

RUSSO, J., BRADLEY, R. H., MCGRATH, C. & RUSSO, I. H. (1977) Transmission electron microscopy study of a human breast carcinoma cell line (MCF7) cultured in collagen-coated cellulose sponge. *Cancer Res.*, **37**, 2004.

SCHULTZ, G. S. & EBNER, K. E. (1977) Alpha-lactalbumin levels in human mammary tumours, sera and mammary cell culture lines. *Cancer Res.*, **37**, 4489.

WOODS, K. L. & HEATH, D. A. (1977) The radioimmunoassay of human lactalbumin. *Clin. Chim. Acta*, **78**, 129.

WOODS, K. L., COVE, D. H., MORRISON, J. M. & HEATH, D. A. (1979) The investigation of lactalbumin as a possible marker for human breast cancer. *Eur. J. Cancer*, **15**, 47.